# 

**Published:** 1853-06

**Authors:** 


### CONTENTS

OF NO VI.

---

### ORIGINAL COMMUNICATIONS.

### ESSAYS AND CASES.

ART. I.—Remarks on Cerebro-Spinal Meningitis, as it prevailed in and near San Augustine, Texas, in the spring of 1852. Submitted to the Trustees and Medical Faculty of the University of Louisville, February 1st, 1852, for the degree of Doctor of Medicine. By Isaiah J. Roberts, M. D. - - - - - - - 565

ART. II.—Milk-Sickness; its Etiology and Treatment. Submitted to the Trustees and Medical Faculty of the University of Louisville, Feb. 1st, 1853, for the degree of Doctor of Medicine. By Samuel W. Thompson, M. D., of Albion, Illinois. - - - - - 471

ART. III.—Case of Disease observed in the Hospitals of Paris. By Elkanah Williams, M. D., of Cincinnati. - 486

ART. IV.—Incised Wound of the Left Side, between the eighth and ninth ribs, followed by protrusion of the Stomach and strangulation of the organ; Reduction and recovery of the patient. By W. W. HART, M. D., of Carrollton, Miss. - - - - - - 496

### REVIEWS.

ART. V.—General Pathology, as conducive to the establishment of rational principles, delivered at St. Thomas's Hospital, during the summer session of 1850. By JOHN SIMON, F. R. S., one of the Surgical Staff of that hospital, and Officer of Health to the city of London. - - 498

ART. VI.—On Syphilis, Constitutional, and Hereditary: and on Syphilitic Eruptions. By ERASMUS WILSON, F. R. S. - - - - - - - 507

SELECTIONS FROM FOREIGN AND HOME JOURNALS.

Death of Dr. Graves. - - - - - 513
Death of M. Orfila. - - - - - 516
The Therapeutic value of Veratrum Viride. - - 519
Asthma—A Clinical Lecture at Hotel Dieu. - - 520
A Case of Poisoning by Arsenious Acid. - - 527
Hats and Baldness. - - - - - - 530
Treatment of Dysentery. - - - - 531
On the Composition of Human Milk in health and disease. 522
Case of Peritonitis with probable Intestinal Perforation. - 535
Case of Strangulation of the Intestine by a Diverticulum Intestini. - - - - - 538
Tartarized Sulphate of Quinine - - - - 540
Dyspepsia. - - - - - - 540

ORIGINAL INTELLIGENCE.

American Medical Association. - - - - 541
The Physicians' Visit to the Islands. - - - 547
Medical Letters from Paris. - - - - 549
Appointments and changes in Medical Schools. - - 550
Health of Louisville. - - - - - - 551
Dr. Marshall Hall. - - - - - - 551
Chloroform in Labor sanctioned by Royal example. - 552
Buffalo Medical Journal. - - - - - 552
Death of Dr. Beaumont. - - - - - 553

